# Peptide Nucleic Acid Probes for MicroRNA Detection: Mg^2+^ Ion Effect, Surface Hybridization, and Surface Plasmon Resonance Biosensing

**DOI:** 10.1021/acsmeasuresciau.5c00100

**Published:** 2025-10-21

**Authors:** Vanessa Jungbluth, Roberta D’Agata, Noemi Bellassai, Stefano Volpi, Roberto Corradini, Giuseppe Spoto

**Affiliations:** † Department of Chemical Sciences, 9298 University of Catania, Viale Andrea Doria 6, 95122 Catania, Italy; ‡ INBB, Istituto Nazionale di Biostrutture e Biosistemi, Via dei Carpegna 19, 00165 Roma, Italy; § Department of Chemistry, Life Sciences and Environmental Sustainability, 9370 University of Parma, Parco Area Delle Scienze, 17/A, 43124 Parma, Italy

**Keywords:** MicroRNA, Peptide nucleic acid, Surface plasmon resonance, Nucleic acids, Biosensors

## Abstract

This study examines how Mg^2+^ ions affect the hybridization between surface-immobilized peptide nucleic acid (PNA) probes and microRNA targets (miR125 and miR141), which is important for the development of nucleic acid-based biosensors utilizing surface plasmon resonance (SPR). The results show that appropriate concentrations of Mg^2+^ significantly enhance microRNA hybridization with PNA probes, whereas Na^+^ does not yield similar results. Kinetic analysis demonstrated that 30 and 100 mM concentrations of Mg^2+^ facilitate the interaction between the PNA probe and its microRNA target by effectively screening the negative charges of the microRNA molecules as they approach the surface. These Mg^2+^ levels also stabilize the heteroduplexes formed on the surface by reducing the dissociation rate. However, a higher Mg^2+^ concentration (300 mM) was found to hinder the surface-confined hybridization. In comparison, Na^+^ showed a considerably smaller role in improving the hybridization. Melting curve analysis in solution indicated that the increase in *T*_m_ of PNA/miRNA heteroduplexes in the presence of Mg^2+^ does not fully explain the enhanced surface interaction, underscoring the role of surface confinement. These findings demonstrate that optimizing the Mg^2+^ concentration can significantly improve the sensitivity and efficiency of PNA- and SPR-based microRNA biosensors. This optimization is particularly relevant for diagnostic and research applications involving the analysis of low concentrations of microRNAs in biofluids.

## Graphical abstract

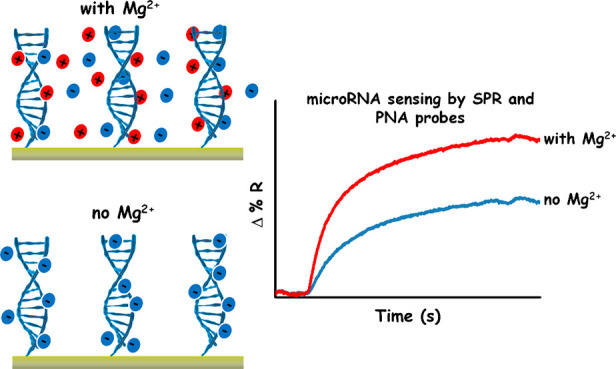

## 1 Introduction

Peptide nucleic acid (PNA) is one of the most popular DNA mimic used in nucleic acid biosensing because of its peculiar physicochemical properties.^1^ Introduced by Nielsen and co-workers in 1991,^2^ PNA exhibits higher stability in biological environments than DNA and RNA due to its resistance to enzymatic degradation.^3^ Its *N*-(2-aminoethyl)­glycine neutral polyamide backbone bestows more robust nucleic acid binding properties than those of natural nucleic acids. PNA/DNA and PNA/RNA heteroduplexes have high thermal stability, even under low ionic strength conditions.^4^ Such stability favors the complexation of complementary genomic sequences^5^ and improves the selectivity of the hybridization and detection of a single mismatch.^6^

The peculiar properties of PNA have allowed expanding applications of nucleic acid-based biosensors.^7,8^ Among them, nucleic acid biosensors relying on surface plasmon resonance (SPR)^9^ with microfluidic sample handling^10^ are advantageous and allow the multiplexed interrogation of biomolecular interactions. As the capture probe in SPR biosensing,^11^ PNAs have been shown to achieve accurate and parallel detection of DNA targets with simplified detection schemes^12^ for the genotyping of single nucleotide polymorphism (SNP),^13^ pathogen detection,^14^ and monitoring of disease-related DNA expression profiles, also in complex biological fluid.^15,16^

PNAs are also promising probes for detecting RNA sequences that are targeted with higher affinity than DNA. The neutral backbone of PNA allows hybridizing the complementary RNA sequence under such low salt conditions as to disfavor the stabilization of RNA secondary structures that hinder easy access to the target sequence.^17^ The mentioned features allow for using short PNA sequences to target RNAs. This aspect may be particularly advantageous in microRNA (miRNA) biosensing^18^ because of miRNA’s small size, sequence homologies, and low concentration in body fluids.^19,20^

miRNAs are increasingly recognized as valuable biomarkers in cancer research due to their pivotal role in cellular communication and gene expression.^21^ However, their detection is challenging due to the above intrinsic features, requiring advanced sensing techniques like SPR. Recent studies showing enhanced sensitivity and multiplexed detection highlight the importance of SPR in developing sensitive diagnostic methods for miRNA detection.^22^

The dependence of PNA/DNA hybridization kinetics on ionic strength has already been investigated.^23^ The hybridization displays a negative salt dependence with increased heteroduplex stability at low ionic strength,^24,25^ unlike the trend for natural nucleic acid duplexes for which increased ionic strength increases the duplex’s melting temperature (*T*_m_). In low ionic strength media, the higher thermodynamic stability of full-matched PNA/DNA heteroduplexes has been partially associated with entropically favorable counterion release upon complexation.^24^ It has also been reported that a several millimolar concentration of Mg^2+^ and a low concentration of Na^+^ provide favorable conditions for DNA detection when using DNA probes.^26^ In the specific case of PNA-RNA complexes in solution, ionic strength plays little effect on hybridization, showing neutral salt dependence.^25^

We focus on surface-bound PNA–miRNA interactions in the presence of Mg^2+^ ions since previous studies have shown that positively charged ions bind to RNA,^27^ playing a role in its stability, simplifying structural complexity and folding that allows RNA molecules to perform various cellular functions.^28^ The higher thermal stability of PNA/RNA heteroduplexes compared to that of PNA/DNA heteroduplexes has been attributed to the A-form structure, which is preferred by RNA under physiological conditions.^29^ This form has been shown to employ tighter and more structured counterion interaction with respect to an intermediate structure between A- and B-form, preferred by PNA/DNA heteroduplexes.^30^

The binding of Mg^2+^ to the negatively charged backbone of nucleic acids in solution has been shown to reduce repulsive forces through charge screening effects. This interaction facilitates the structural reorganization of the strands, allowing them to adopt more compact conformations^31^ and stabilizing duplex formations.^32^ However, an excess of Mg^2+^ can lead to repulsive interactions and potential destabilization or misfolding of nucleic acid structures, counteracting the stabilizing effects.^33^ This highlights the complex nature of ion interactions within nucleic acid systems.

The literature indicates that there are complex effects between monovalent and divalent cations, which complicate the prediction of DNA/RNA stability under various buffer conditions and demonstrate the necessity for optimal ionic conditions.^34,35^ Additionally, the relationship between duplex morphology and orientation on surfaces, as well as how ions affect the formation of heteroduplexes between unmodified PNA and RNA targets, remains poorly explored but is crucial for understanding nucleic acid surface hybridization.

The nucleic acid hybridization on a surface can deviate considerably from the models confirmed in solution^36^ because of the multitude of variables involved in the surface-confined process.^37^ Previous studies examining the ionic effect on surface PNA/DNA hybridization^38^ have primarily focused on probe density^39,40^ and the influence of different types and concentrations of cations.^26,41–43^

However, there are currently no available data regarding the specific influence of ions on PNA/RNA surface hybridization.

It is well established that high-performance nucleic acid sensors require sensitive and efficient probe–target interaction, which depends greatly on the surface architecture,^44^ probe density, and buffer composition.^45^

We investigated the influence of Mg^2+^ and Na^+^ ions on the hybridization of miRNA targets at different concentrations to complementary surface-immobilized PNA probes. The influence of these cations on surface hybridization is examined in relation to both the target concentration and the type and concentration of the cations used. This study has been conducted using an SPR imaging (SPRI) apparatus to investigate surface-confined interactions. The results are then compared to those obtained in solution by utilizing optimized buffer conditions to analyze the melting temperatures of PNA/miRNA heteroduplexes. This comparison aims to show that magnesium ions enhance the formation of PNA/miRNA heteroduplexes on the surface, thus improving the ability of SPRI to detect miRNA targets.

## 2 Experimental Section

### 2.1 Materials and Reagents

All reagents were obtained from commercial suppliers and used without further purification. Dimethyl sulfoxide (DMSO), dithiobis­(*N*)­succinimidylpropionate (DTSP), ethanol (EtOH), magnesium chloride (MgCl_2_), sodium bicarbonate (NaHCO_3_), sodium chloride (NaCl), sodium hydroxide (NaOH), sodium dodecyl sulfate (SDS), Tris-hydrochloric acid, and urea were purchased from Merck (Italy). Phosphate buffered saline (PBS) solutions at pH 7.4 (137 mM NaCl, 2.7 mM KCl, phosphate buffered 9.55 mM) were obtained from VWR (Italy). The PNA probe for miR125 (PNA-miR125) was obtained from Panagene (Korea), whereas the PNA probe for miR141 (PNA-miR141) was produced using automatic solid-phase synthesis,^46^ purified by HPLC, and characterized by UPLC-MS, as described elsewhere,^47^ for purity and identity assessment (see Figure S8 for full characterization). PNA-miR141 was also used as the control probe when performing experiments aimed at investigating the PNA-miR125 interaction with the miR125 target. miR125 (MW = 6842 Da) and miR141 (MW = 7070 Da) were purchased from Eurofins (Italy) and IDT (Italy), respectively. Each miRNA sample was split into small aliquots and stored at −80 °C upon arrival. All miRNA stock solutions were prepared daily with nuclease-free water (Ambion, Autoclaved, 0.2 μm, filtered) in an RNase-free environment. SPRI gold chips were purchased from XanTec Bioanalytics GmbH (Germany). Ultrapure water (Integral S3, Millipore) was used for all the experiments.

### 2.2 miRNA Targets and PNA Probe Design

We selected miR125 and miR141 as the targets for this study. These miRNAs were found to be downregulated in serum and tissue samples of glioma patients and are considered diagnostic biomarkers for gliomas.^48,49^ The subtypes selected for this study include hsa-mir-125b-5p, which is an isoform derived from the 5′ arm of premir-125b, and hsa-mir-141-3p, which is a member of the miR200 family. The miR125 family consists of three homologous members: hsa-mir-125a, hsa-mir-125b-1, and hsa-mir-125b-2. These microRNAs are known to be involved in cell differentiation, proliferation, and apoptosis. On the other hand, the miR200 family includes five subfamilies: miR200a, miR200b, miR200c, miR141, and miR429. miR141 shares certain characteristics with the miR125 family. Both microRNAs can play context-dependent roles as either tumor suppressors or oncogenes in various diseases, particularly in cancer and immune responses.^50,51^

A PNA sequence complementary to the miR125 target (PNA-miR125) was designed. It was modified with two (2-(2-aminoethoxy)­ethoxy)­acetyl (AEEA) linkers, serving as a spacer for surface attachment and minimizing surface effects caused by the steric hindrance of immobilized systems. A PNA sequence complementary to the target miR141 (named PNA-miR141), modified with two AEEA linkers, was designed, synthesized, and used for SPRI additional experiments and melting curve studies. All sequences used in this study are noted in Table 1.

**Table 1:** Sequences and Acronyms Used in This Study^a^

Acronym	Sequence^a^
PNA-miR125	Ac-AAG TTA GGG TCT CAG GGA-Lys(AEEA)_2_-NH_2_
PNA-miR141	H-(AEEA)_2_-CCA TCT TTA CCA GAC AGT-Gly-NH_2_
miR125	UCC CUG AGA CCC UAA CUU GUG A
miR141	UAA CAC UGU CUG GUA AAG AUGG

a Underlined letters identify miRNA sequences complementary to the corresponding PNA probe.

b (AEEA): (2-(2-aminoethoxy)­ethoxy)­acetyl spacer at C-term or N-term.

Melting temperatures of PNA/DNA duplex sequences were estimated using the PNA-BIO software PNA tool, which predicts thermal stability for the corresponding duplex by employing a modified version of the nearest neighbor model. These estimates (Table S1) were considered indicative, as PNA/RNA melting temperatures are typically higher than those predicted for corresponding DNA sequences under similar conditions.^52^

### 2.3 SPRI Apparatus and Measurements

Detailed descriptions of the SPRI experimental setup and data analysis methods have been given in earlier works^53,54^ and are summarized in the Supporting Information.

### 2.4 PNA Probe Immobilization and miRNA Hybridization

PNA probes were immobilized on DTSP-modified gold SPR chips through the amine-coupling reaction between *N*-hydroxysuccinimidyl (NHS) ester ends of DTSP and AEEA linker, found on either the C-terminal or N-terminal groups of the PNA probes. The spatially separated immobilization of PNA probes was obtained by injecting PNA solutions (0.1 μM in PBS, flow rate of 10 μL min^–1^ for 30 min) into parallel microchannels in contact with the DTSP-modified gold surface. After the probe immobilization and equilibration with PBS buffer, the modified surface was passivated with Trizma (0.5 M in RNase-free water for 10 min) to block the still reactive NHS groups.

SPRI miRNA hybridization experiments were performed at room temperature by using 500 μL of 50, 100, and 250 nM miR125 in PBS buffer added with 0, 0.3, 30, 100, and 300 mM MgCl_2_ or 0, 0.6, 60, 200, and 600 mM NaCl. Solutions were adsorbed (flow rate of 10 μL min^–1^ for 30 min) on surfaces functionalized with a specific or control PNA sequence.

### 2.5 Fitting Routines

All the fits of the provided data sets were obtained using the R nonlinear least-squares (nls) routine, according to a 1:1 kinetic binding model,^55,56^ through an open-source online Anabel software tool.^57^ *k*_obs_ is defined as the observed binding rate constant and reflects the curvature of the calculated fitting curve. Here, all binding rate constants are calculated from a single-curve binding analysis, which must contain an association part as well as a dissociation part. Specifically, *k*_obs_ is defined as *k*_obs_ = *k*_on_[*A*] + *k*_off_, where *k*_on_ refers to the association binding rate constant, *k*_off_ is the dissociation binding rate constant, and [*A*] is the analyte concentration. In the case of the dissociation curve fit, *k*_on_ is assumed to be zero, which leads to *k*_obs_ equaling *k*_off_. The affinity constant *K*_A_ can be calculated from *k*_off_, *k*_obs_, and [*A*] as *K*_A_ = (*k*_obs_ – *k*_off_)/(*k*_off_[*A*]). To fit the sensorgrams used to evaluate the role of Mg^2+^, *t*_on_ and *t*_off_ were imposed as 190 and 2100 s, respectively, while *t*_on_ and *t*_off_ were equal to 200 and 1900 s for kinetic curves recorded to asses the impact of Na^+^ on PNA–miRNA interaction.

### 2.6 Melting Curve Analysis

Measurements were performed on an Agilent Cary 3500 instrument equipped with a Compact Peltier temperature controller unit or on a Jasco J715 spectropolarimeter (Tokyo, Japan) equipped with a Peltier PTC 348 temperature controller unit. CD spectra were obtained by using a scan speed of 100 nm/min and three accumulations for each spectrum. The spectra were corrected by subtracting the signal obtained from the corresponding buffer. The melting analyses of the PNA-miR141/miR141 heteroduplexes were conducted at the wavelength where the maximum difference between the RNA and PNA/RNA spectra was observed, specifically 260 nm. Quartz cells with a path length of 1 cm were used, and the sample volume was 500 μL. Solutions containing PNA (4 μM) and complementary miRNA (4 μM) were prepared by diluting their aqueous stock solutions with PBS buffer (both in the absence and in the presence of 0.3, 30, and 100 mM MgCl_2_). The solutions were incubated at 95 °C for 5 min, followed by gradual cooling to room temperature. The final solution (2 μM) was transferred to the cuvette for analysis. Melting curves were recorded by measuring the absorbance at 260 nm as the temperature was varied from 25 to 90 °C. Data were collected at 1° intervals with a ramp rate of 1 °C/min. *T*_m_ was determined as the maximum of the first derivatives of the melting curves using the Cary UV Workstation software.

## 3 Results and Discussion

### 3.1 SPR Analysis of the Hybridization between PNA Probes and miRNA

It is generally proposed that the uncharged backbone of PNA minimizes electrostatic repulsion, thereby enhancing the hybridization efficiency with negatively charged nucleic acid targets and facilitating stronger interactions with DNA and RNA sequences. This work highlights the importance of electrostatic interactions by investigating the effects of added salts (Mg^2+^, Na^+^) and assessing their contribution to improving hybridization between PNA and microRNA.

Although the benefits of using PNA as probes are well documented, the local concentration of PNA, which is influenced by its surface density, directly affects the SPR sensing capacity. An increased probe density can enhance signal transduction and improve analytical performance;^58^ however, it may also lead to steric hindrance, reducing target accessibility to the probes. Achieving a balance between these factors is critical for optimizing biosensor design.

The surface density and orientation of PNA probes are known to affect target hybridization efficiency and may impose critical constraints on the reaction. In densely packed PNA layers, the electrostatic repulsion between incoming DNA and RNA targets can hinder hybridization. The repulsion can be mitigated by modifying the hybridization buffer ionic strength or by reducing the probe density. Such procedures may ensure that complementary strands hybridize through a straightforward pseudo-first-order kinetics process. To achieve an optimal density of PNA probes, it is essential to perform controlled surface immobilization. This approach minimizes steric cross-talk between neighboring PNA probes and reduces electrostatic repulsions with incoming miRNA targets during hybridization.

We immobilized PNA-miR125 and PNA-miR141 probes (Table 1) on the SPRI gold sensor surface through a covalent coupling reaction. The reaction involved the amine moiety present in the spacer of the PNA probe and the NHS groups on the functionalized gold surface.

Figure 1a shows the Δ%*R* over time detected for the spatially separated surface immobilization of PNA-miR125 and PNA-miR141 probes. The experiments were conducted using 0.1 μM probe solutions. Control experiments assessing the interaction between miR125 and the complementary PNA-miR125 probe were conducted using the PNA-miR141 probe, which is not related to miR125. An increase in Δ%*R* was observed upon injection of PNA solutions, which, after 30 min, were replaced with PBS buffer to wash off any unbound molecules for 10 min. Using the average value obtained from replicated experiments aimed at detecting the SPRI signal after the immobilization of the PNA probe (PNA-miR125, Δ%*R* = 3.340.97, *n* = 52), we quantified the layer thickness and surface coverage. The thickness was determined to be 0.5 nm, while the surface coverage was calculated to be 5.6 × 10^12^ molecules cm^–2^. These calculations were based on the theoretical model described by Shumaker-Parry.^59^

**Figure 1:**
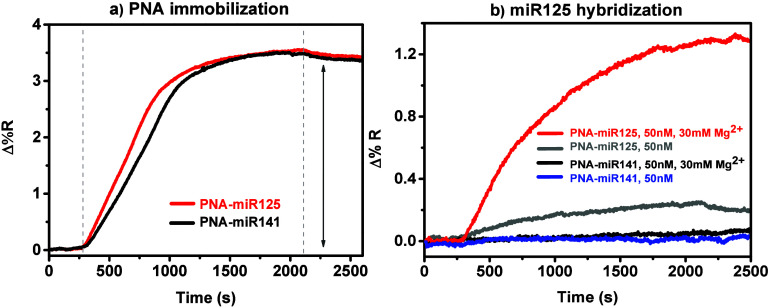
(a) Representative changes in percent reflectivity (Δ%*R*) over time for the immobilization of PNA-miR125 (red line) and PNA-miR141 (black line) (0.1 μM in PBS) onto a DTSP-modified gold surface. (b) Δ%*R* over time for the hybridization between miR125 (50 nM in PBS with and without 30 mM Mg^2+^) and PNA-miR141 (acting as the control) or PNA-miR125.

Efficient hybridization between the probe and the target has been established in line with previous works,^12,60,61^ indicating that surface coverages in the range of 2 × 10^12^ to 9 × 10^12^ molecules cm^–2^ are optimal. In particular, the obtained moderate probe surface density is appropriate for achieving good hybridization efficiency^62^ and a uniform immobilization level of the PNA probe on the surface.

After considering the surface density of the PNA probe, we evaluated the its capacity to detect miR125 by identifying the best conditions for the interaction. A 50 nM solution of miR125 in PBS was allowed to interact with the specific and control surface-immobilized PNA probes (PNA-miR125 or PNA-miR141, respectively) in parallel. The control experiments conducted to assess nonspecific interactions of miR125 with an unrelated PNA probe (PNA-miR141) resulted in an SPRI signal close to the instrumental noise (Figure 1b, blue line). The specific interaction of a 50 nM miR125 solution with PNA-miR125 generated a moderate intensity SPRI response (Figure 1b, gray line), and experiments conducted with solutions of varying concentrations yielded concentration-dependent SPRI responses (Figure S1). The measured Δ%*R* values, along with the calculated average surface coverage and hybridization efficiency (HE) for the specific interaction with PNA-miR125, are reported in the Supporting Information (Table S2).

HE refers to the percentage of surface-immobilized PNA probes that hybridize with the miRNA target. As reported elsewhere, it is calculated as the interaction stoichiometry, which equals the target surface coverage divided by the probe surface coverage.^26,62^

Mg^2+^ ions have been reported to bind oligobases,^63^ thus potentially leading to a surface interaction with noncomplementary sequences. We observed a negligible SPRI signal when a 50 nM solution of miR125 was added with 30 mM Mg^2+^ and used to assess the level of nonspecific adsorption on the unrelated PNA-miR141 probe (Figure 1b, black line). Such evidence suggests that Mg^2+^-mediated interchain interactions, apart from heteroduplex formation, are unlikely during surface hybridization processes with PNA probes. The experiments also show that the increased SPRI response detected specifically for the interaction between miR125 and PNA-miR125 in the presence of 30 mM Mg^2+^ cannot be attributed to the extra mass resulting from the surface coadsorption of Mg^2+^ ions.

We utilized SPRI to evaluate the interaction between PNA-miR125 and its complementary target miR125 by introducing MgCl_2_ into the hybridization buffer at a concentration that closely resembles Mg^2+^ physiological conditions.^64^

Adding 30 mM MgCl_2_ into the hybridization buffer significantly enhanced the sensitivity for detecting the adsorption of 50 nM miR125 on immobilized PNA-miR125 (Figure 1b, red line). We measured an average SPRI response of Δ%*R* = 0.3 ± 0.1 for 50 nM miR125 without Mg^2+^, compared to Δ%*R* = 1.1 ± 0.1 when the hybridization occurred in PBS buffer supplemented with 30 mM Mg^2+^.

HE percentages for the immobilized PNA-miR125 probe and 50 nM miR125 significantly increased when the hybridization was conducted with 30 mM MgCl_2_ in the buffer. Specifically, the HE improved from 16% in PBS to 56% in PBS with 30 mM Mg^2+^ (see Table S2).

### 3.2 Effect of Mg^2+^ on PNA Probe–miRNA Surface Interaction

After establishing that Mg^2+^ ions enhance the ability of the surface-immobilized PNA-miR125 probe to form a heteroduplex with the target miR125, we proceeded to investigate the direct effect of introducing Mg^2+^ at various concentrations into the hybridization buffer on the extent of PNA/miRNA hybridization, as measured by SPRI. We calculated HE percentages after immobilizing the PNA-miR125 probe (0.1 μM) and hybridizing it with miR125 targets at various concentrations (50, 100, and 250 nM). We utilized our SPRI platform to simultaneously conduct these surface hybridization reactions using separate flow channels in contact with the SPRI gold sensor surface.

As mentioned earlier, standard nucleic acid hybridization protocols require salt concentrations whose effect on DNA and RNA molecules apply through nonspecific electrostatic interactions, which depend on the ionic strength of the medium.^65^ The mechanism by which ions influence nonmodified PNA heteroduplex formation remains poorly explored for RNA target interaction.

To prove that the Mg^2+^ plays a role in the hybridization reaction between miRNA and PNA probes by improving the hybridization process, a concentration series of miR125 (50, 100, and 250 nM in PBS) enriched with Mg^2+^ (0.3, 30, 100, and 300 mM) was allowed to interact with the surface-immobilized PNA-miR125. The data presented in Figures 2a and 3a demonstrate that 0.3 mM Mg^2+^ in PBS buffer has a negligible effect on the hybridization reaction when compared to PBS buffer alone (Figure S2), also resulting in similar HE percentages (HE = 16%, 16%, and 34% for 50, 100, and 250 nM miR125 with only PBS vs HE = 14%, 22%, and 30% for 50, 100, and 250 nM miR125 with 0.3 mM Mg^2+^ in PBS; Figure 3b and Table S2).

**Figure 2:**
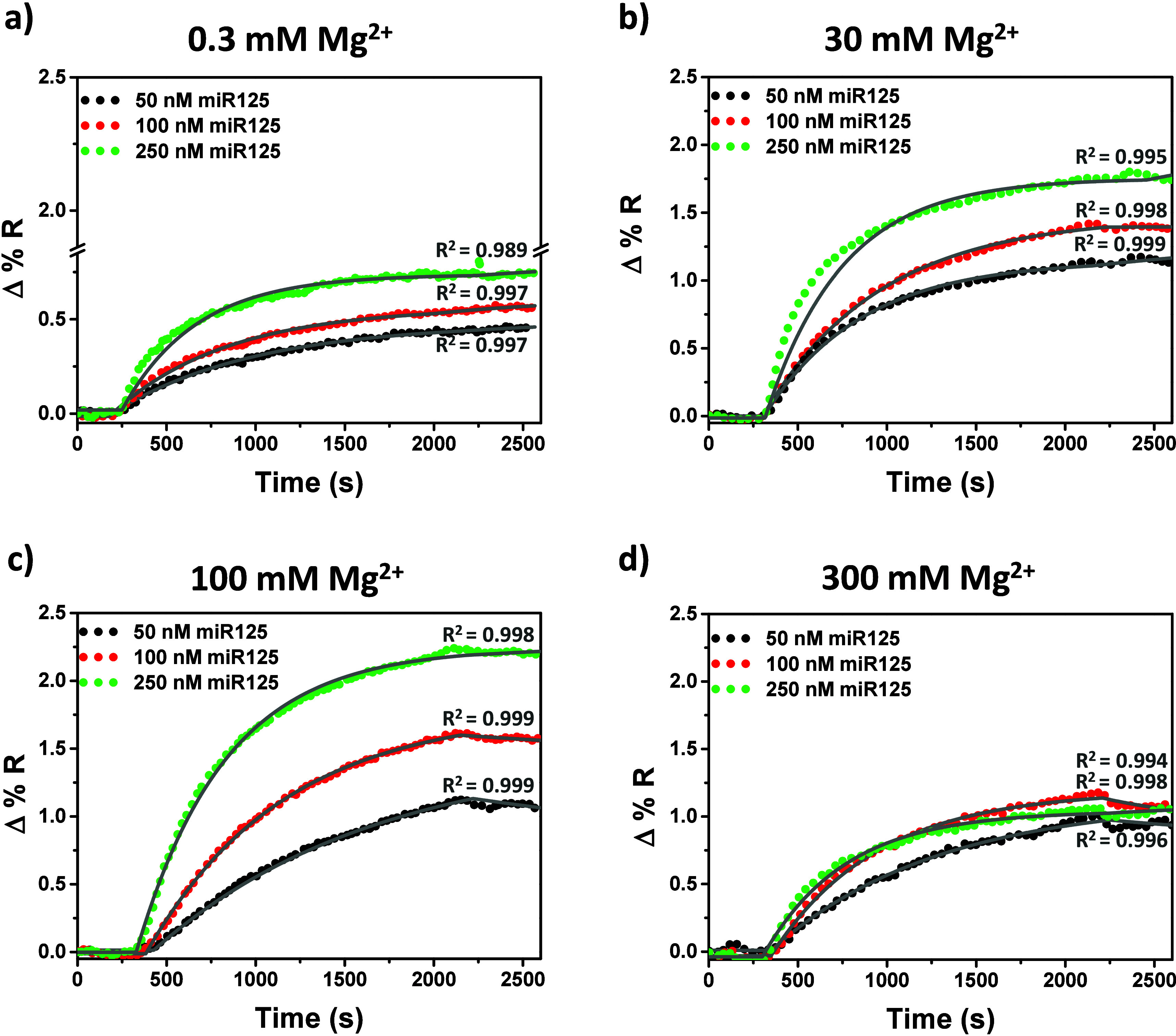
SPRI curves for the interaction of PNA-miR125 with miR125 solutions at concentrations of 50 (black dots), 100 (red dots), and 250 nM (green dots) in PBS buffer, which was supplemented with varying Mg^2+^ levels (0.3, 30, 100, and 300 mM). The gray lines represent the results of the nonlinear regression used to estimate the kinetic data presented in Table 2. *R* ^2^ values for each regression are also provided.

**Figure 3:**
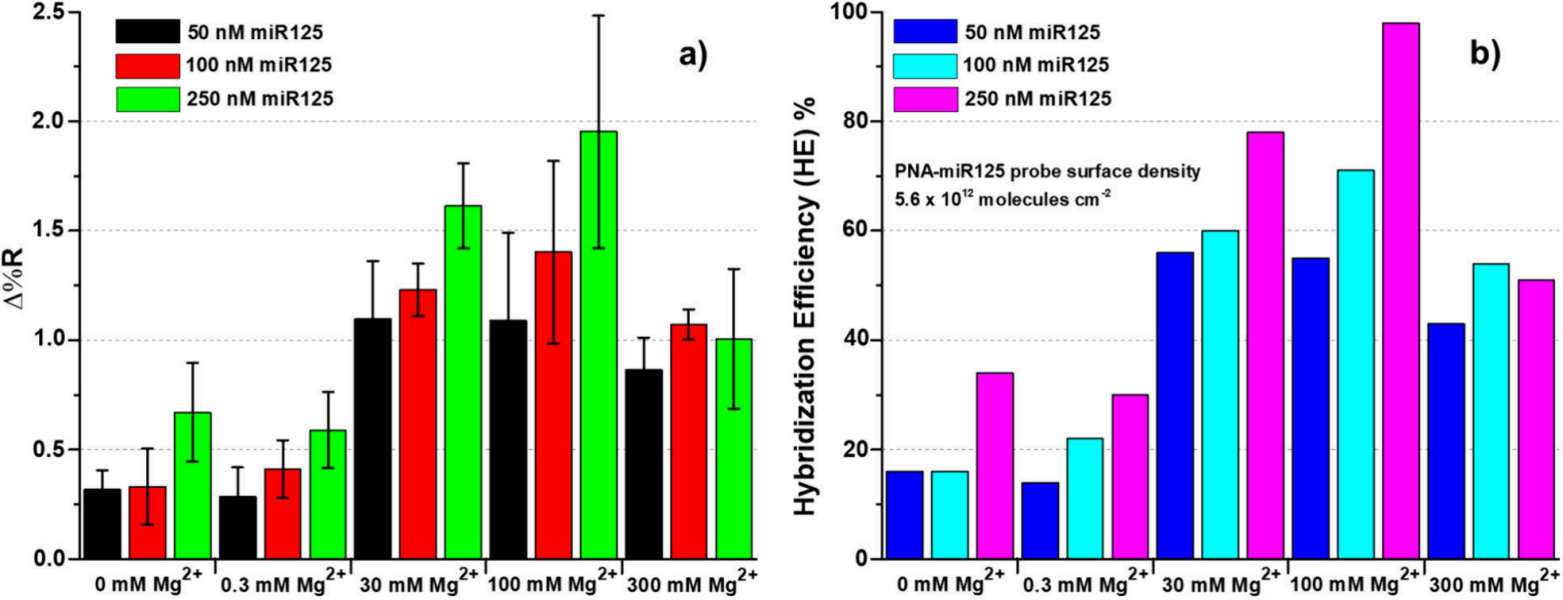
(a) Average Δ%*R* values detected for the hybridization reaction between immobilized PNA-miR125 and miR125 at different concentrations (50, 100, and 250 nM in PBS) with varying Mg^2+^ concentrations in PBS (0, 0.3, 30, 100, and 300 mM). Error bars represent the standard deviation (*n* > 3). (b) Hybridization efficiency (HE) values calculated as the interaction stoichiometry, equal to target surface coverage/probe surface coverage, with varying Mg^2+^ concentrations in PBS (0, 0.3, 30, 100, and 300 mM). The plotted numerical values are reported in Table S2.

Higher concentrations of Mg^2+^ (30 and 100 mM) significantly enhanced the SPRI-detected signals for miR125 hybridization (Figures 2 and 3, Table S2). HE also significantly increased, achieving HE = 98% for the interaction of 250 nM miR125 in the presence of 100 mM Mg^2+^. Further increasing the Mg^2+^ concentration to 300 mM partially suppressed the detected signal and HE percentages.

Mg^2+^ concentration also affected the SPRI sensitivity in detecting miR125 using an immobilized PNA-miR125 probe. As shown in Figure 4, 0.3 mM Mg^2+^ in PBS did not change the SPRI sensitivity obtained with PBS alone (*S*_0 mM Mg^2+^_ = 1.88 ± 0.39 × 10^–3^ Δ%*R*/ΔConc. (nM) vs *S*_0.3 mM Mg^2+^_ = 1.44 ± 0.25 × 10^–3^ Δ%*R*/ΔConc. (nM)). Both the 30 and 100 mM concentrations of Mg^2+^ significantly enhanced the assay sensitivity, with the highest sensitivity observed when 100 mM Mg^2+^ was present during the PNA-miR125/miR125 surface hybridization (*S*_30 mM Mg^2+^_ = 2.28 ± 0.12 × 10^–3^ Δ%*R*/ΔConc. (nM), *S*_100 mM Mg^2+^_ = 4.15 ± 0.50 × 10^–3^ Δ%*R*/ΔConc. (nM)). Interestingly, a further increase in Mg^2+^ concentration to 300 mM dramatically affected the assay sensitivity, creating the least favorable conditions for PNA-miR125/miR125 surface hybridization, even compared to experiments conducted without Mg^2+^ (*S*_300 mM Mg^2+^_ = 0.43 ± 0.91 × 10^–3^ Δ%*R*/ΔConc. (nM)).

**Figure 4:**
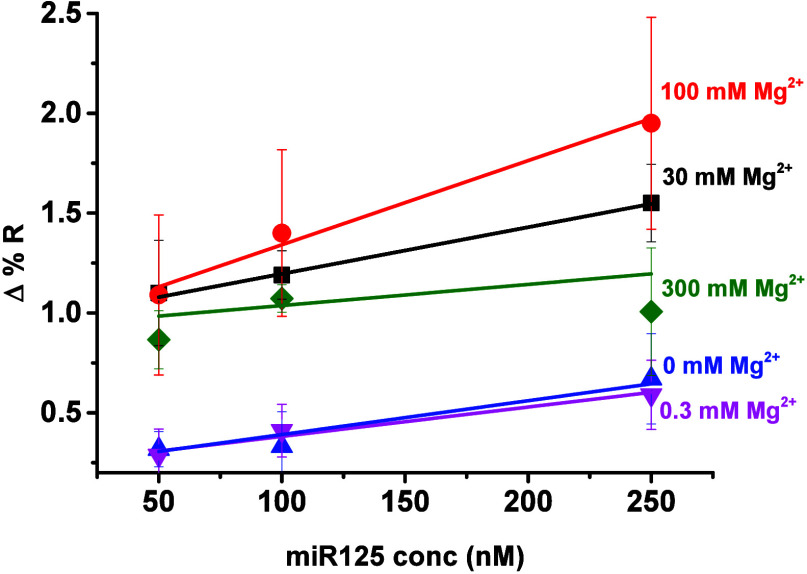
Δ%*R* values detected for the hybridization reaction involving immobilized PNA-miR125 and miR125 at various target concentrations (50, 100, and 250 nM in PBS) and differing Mg^2+^ concentrations in PBS (0, 0.3, 30, 100, and 300 mM).

We interpret the dependence of the SPRI response on the Mg^2+^ concentration as a direct result of the electrostatic interaction between the negatively charged miR125 target strands and Mg^2+^ ions. While PNA is neutral, the PNA/miRNA heteroduplex acquires a partial negative charge because of the hybridized miRNA. Mg^2+^ ions act as a stabilizing factor for the interaction between PNA and miR125, helping to counteract the decreased accessibility of miR125 on the surface that results from interactions between the miRNA strands. This reduced accessibility occurs due to the electrostatic repulsion between incoming miRNA strands and those already hybridized with the PNA probe.

Mg^2+^ may influence the specific interaction between PNA and miRNA due to its ability to bind to the PNA polyamide backbone and nucleobases. This involvement potentially includes the N7 sites of purines and the N3 sites of pyrimidines.^43^ Our preliminary experiments assessed the nonspecific adsorption of miR125 on unrelated PNA-miR141 probes, and the results showed that Mg^2+^ does not introduce artifacts that might affect the evaluation of the specificity of the assessed interaction. However, we cannot exclude the possibility that Mg^2+^ interacts with the polyamide backbone and nucleobases during the specific surface hybridization of PNA-miR125 with miR125.

Based on the data presented so far, 100 mM Mg^2+^ establishes the most favorable conditions for the interaction, enhancing the detected SPRI signal and improving the assay sensitivity. The negative effects of a high Mg^2+^ concentration (300 mM) may result from excessive positive charge accumulation, which can reverse the electrostatic driving force and make the adsorption of miRNA less favorable. Additionally, such a high concentration of Mg^2+^ might affect the conformation of incoming miR125, making it less likely to hybridize with the PNA-miR125 probe.

We compared the previously described effects of Mg^2+^ on PNA-miR125/miR125 hybridization with those of Na^+^. To achieve this, we created a concentration series of miR125 solutions (50, 100, and 250 nM in PBS) supplemented with Na^+^ at concentrations of 0.6, 60, 200, and 600 mM. We doubled the concentration of Na^+^ in comparison to the corresponding Mg^2+^ solutions to ensure that the total charge concentration remained consistent across both sets of solutions. This approach helps emphasize the potential role of the 5-fold higher charge density of Mg^2+^ compared to that of Na^+^.

Figure 5 displays the SPRI signals detected after the hybridization of miR125 across the various surface interactions investigated (see also Figure S3). A comparison of these results with those obtained using Mg^2+^ (Figure 2) indicates that Na^+^ plays a significantly diminished role in the hybridization of miR125 with surface-immobilized PNA-miR125. Interestingly, a Na^+^ concentration of up to 60 mM has no effect on the intensity of the SPRI-detected signals after the hybridization process. In comparison, 200 mM Na^+^ enhances the detected SPRI signals, albeit still with lower intensity than those detected with 100 mM Mg^2+^.

**Figure 5:**
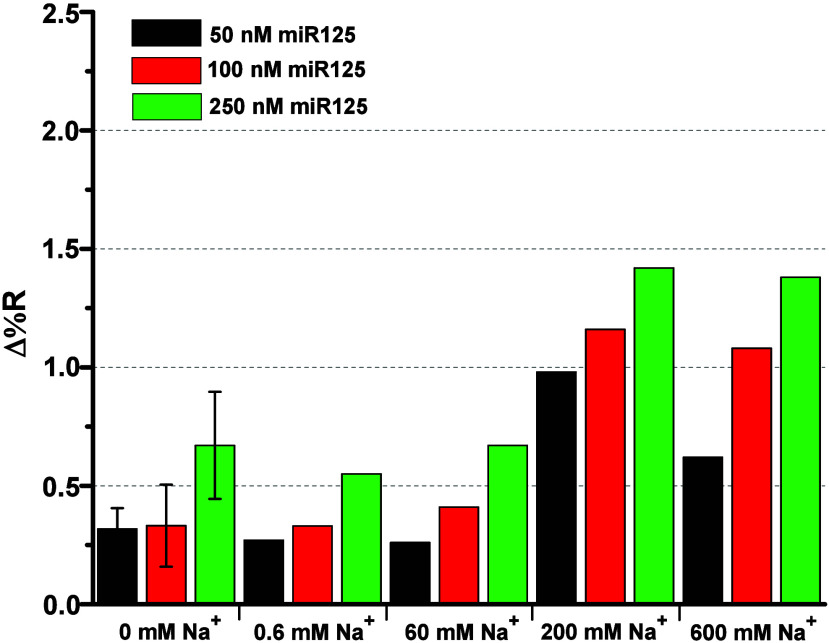
Average Δ%*R* values detected for the hybridization reaction between immobilized PNA-miR125 and miR125 at different concentrations (50, 100, and 250 nM in PBS) with varying Na^+^ concentrations in PBS (0, 0.6, 60, 200, and 600 mM).

Table 2 presents the association and dissociation rate constant values (*k*_on_ and *k*_off_, respectively) for the different surface hybridization reactions (see also Figure 3 and Figure S3). Both Mg^2+^ and Na^+^ impact the association rate constant values. At concentrations ranging from 30 to 100 mM, Mg^2+^ enhances the association between miR125 and the PNA-miR125 probe more effectively than Na^+^, which is tested at concentrations between 60 and 200 mM. This effect may result from the screening of the negatively charged backbone of the miR125 targets as they approach the PNA-miR125-modified surface. Both positive ions enhance this screening, with Mg^2+^ being more effective than Na^+^ because of its higher charge density.

**Table 2:** Kinetic Data for the Different Surface Hybridization Reactions Performed with Mg^2+^ or Na^+^ ^a^

Ion Concentration (mM)	*k*_on_ × 10^3^ (M s)^−1^	*k*_off_ × 10^–3^ (s^–1^)	*K*_A_ × 10^6^ (M^–1^)
[Mg^2+^]			
0	2 ± 2	1.0 ± 0.5	3 ± 2
0.3	4 ± 1	1.0 ± 0.1	5 ± 1
30	11 ± 3	0.6 ± 0.2	19 ± 3
100	9 ± 2	0.37 ± 0.04	34 ± 2
300	2.5 ± 0.6	1.2 ± 0.3	2.0 ± 0.6
[Na^+^]			
0	2 ± 2	1.0 ± 0.5	3 ± 2
0.6	6 ± 2	1.6 ± 0.3	3 ± 2
60	8 ± 3	1.5 ± 0.2	6 ± 3
200	5 ± 3	1.2 ± 0.3	5 ± 4
600	4.1 ± 0.9	1.2 ± 0.2	3.5 ± 0.9

a *k*_on_ and *k*_off_: association and dissociation rate constants, respectively. *K*_A_: affinity constant.

Examining the trends in dissociation rate constants reveals interesting insights related to various concentrations of Mg^2+^ and Na^+^. Na^+^ has a moderate effect on the dissociation rate of miR125 from the PNA-miR125/miR125 heteroduplex formed on the surface of the SPRI sensor, with values ranging from *k*_off_ = 1.0 ± 0.5 × 10^–3^ M^–1^ to *k*_off_ = 1.6 ± 0.3 × 10^–3^ M^–1^. In contrast, Mg^2+^ plays a different and more significant role. 100 mM Mg^2+^ reduces the dissociation rate by approximately a factor of 4. Specifically, the dissociation rate (*k*_off_) is measured at 0.37 ± 0.04 × 10^–3^ M^–1^ with 100 mM Mg^2+^, compared to 1.0 ± 0.5 × 10^–3^ M^–1^ with 0 mM Mg^2+^. This higher concentration of Mg^2+^ stabilizes the PNA-miR125/miR125 heteroduplex on the surface much more effectively than Mg^2+^ solutions at other concentrations and is significantly more efficient than any Na^+^ solution (Table 2). Similar conclusions can be drawn from evaluating the trends observed in *K*_A_ values. 30 and 100 mM Mg^2+^ concentrations produce the highest affinity interactions when compared to solutions with different Mg^2+^ levels. In particular, 100 mM Mg^2+^ significantly stabilizes the PNA-miR125–miR125 interaction, resulting in an affinity constant that is more than five times higher than those calculated for interactions conducted with any of the investigated Na^+^ concentrations.

Based on the data presented so far, a concentration of 100 mM Mg^2+^ offers the optimal balance. This concentration enhances the association rate due to the screening effect of the negative charge on the miR125 molecules as they approach the SPRI sensor surface. It reduces the dissociation rate by stabilizing the surface-bound heteroduplexes.

To further support the role of Mg^2+^ in favoring PNA-miR125/miR125 surface hybridization, we conducted experiments to exclude the possibility that the observed signal enhancement was due to changes in ionic strength from increased MgCl_2_ concentrations. To achieve this, we conducted experiments using buffers at constant ionic strengths with varying concentrations of NaCl and MgCl_2_. The optimal background ionic strength for an SPR experiment varies depending on the system being studied. It is well established that both intracellular and extracellular ionic strengths are significantly lower than 1 M for many biological processes. SPR experiments are therefore often performed at lower than 1 M ionic strength to better mimic physiological conditions.^66^

As already pointed out, the best sensing performance for miR125 occurred when 100 mM Mg^2+^ was present in the PBS buffer during the surface hybridization of PNA-miR125 with miR125, achieving an ionic strength of 0.465 M (Figure 2c). We then conducted SPRI experiments using PBS buffers with 0, 0.3, 30, and 100 mM MgCl_2_. We also adjusted the ionic strength to the desired final value of 0.465 M using NaCl. Such a protocol enabled us to assess the different impacts of Na^+^ and Mg^2+^ ions on PNA-miR125/miR125 surface hybridization by excluding any variability associated with the different ionic strengths.

Figure 6 shows SPRI curves illustrating the hybridization reaction between the surface-immobilized PNA-miR125 probe and miR125 at concentrations of 50, 100, and 250 nM in PBS, with varying concentrations of Mg^2+^ and Na^+^ while maintaining a constant ionic strength.

**Figure 6:**
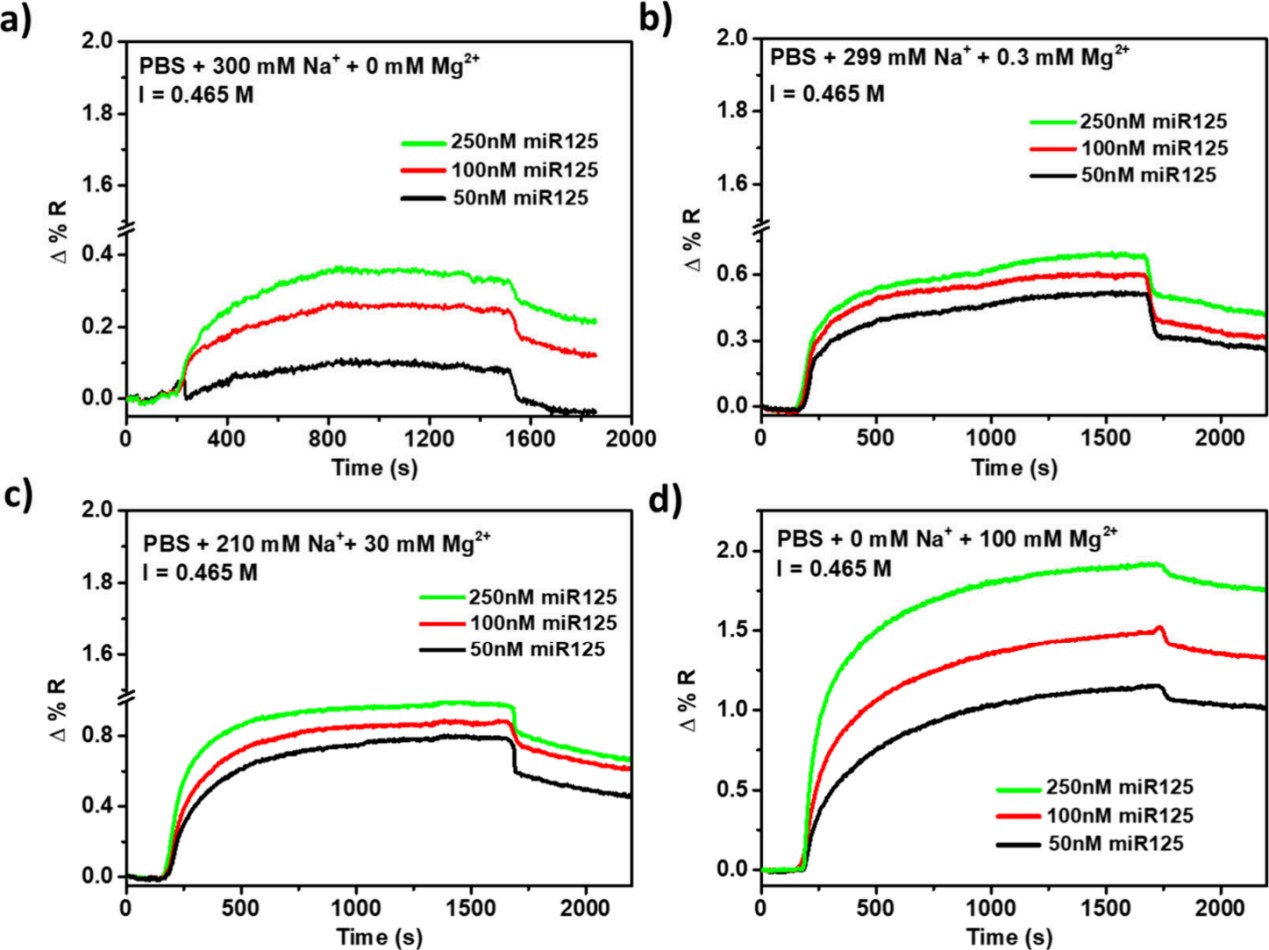
SPRI curves showing PNA-miR125 hybridization with miR125 at 50 (black line), 100 (red line), and 250 nM (green line) in PBS buffer at constant ionic strength (*I* = 0.465 M) with varying Mg^2+^ and Na^+^ concentrations.

The results presented in Figure 6 highlight the peculiar role of Mg^2+^ in comparison to Na^+^. Specifically, comparing panels a and d of Figure 6 allows for a direct assessment of the intensity of the SPRI signal detected when the same ionic strength is obtained using only Na^+^ (Figure 6a) or Mg^2+^ (Figure 6d). Notably, the use of Mg^2+^ yields a more than 5-fold increase in the detected signal.

To demonstrate that the impact of Mg^2+^ on the SPRI detection of miR125 hybridization with immobilized PNA-miR125 probes extends to other PNA–miRNA interactions, we assessed the hybridization of miR141 with the immobilized PNA-miR141 probe using SPRI. This confirmed that Mg^2+^ enhances the SPRI signal, even when the PNA–miRNA interaction exhibits a different orientation and nucleobase composition for PNA (Figure S4).

### 3.3 Effect of Mg^2+^ on PNA–miRNA Interaction in Solution

The experiments presented explored how Mg^2+^ affects the interaction between miRNAs and surface-immobilized PNA probes. It is crucial to further understand whether the confinement of this interaction on the surface contributes to the significant signal enhancement observed in the presence of Mg^2+^ at concentrations of 30 and 100 mM. With this aim, we evaluated how the *T*_m_ of PNA/miRNA heteroduplexes depends on Mg^2+^ concentration. These experiments focus on PNA/miRNA hybridization reactions in solution, thus excluding any effects related to the confinement of the reaction on a surface.

Attempts to determine *T*_m_ of the PNA-miR125/miR125 heteroduplex by measuring its absorbance at 260 nm were unsuccessful (Figure S5). Even at the highest temperature tested (96 °C), the data displayed a sigmoidal trend, and the midpoint of the curve had not yet been reached, regardless of whether Mg^2+^ was present. CD spectra recorded at both 25 and 90 °C showed the formation of a complex with a typical PNA/RNA duplex signature (Figure S6).^67^ We concluded that the *T*_m_ of the PNA-miR125/miR125 heteroduplex exceeds 90 °C, in both the presence and absence of Mg^2+^. This finding is consistent with the calculated *T*_m_ of 83.4 °C for the DNA/PNA duplex, considering that RNA/PNA duplexes exhibit a higher *T*_m_.

In the absence of definitive evidence regarding the influence of Mg^2+^ on *T*_m_ of the PNA-miR125/miR125 heteroduplex, it was essential to validate the entire hypothesis using an alternative PNA/miRNA heteroduplex with a measurable *T*_m_. For this reason, to evaluate the effect of Mg^2+^ on the interaction between PNA and microRNA in solution, we also evaluate the thermal stability of the PNA- miR141/miR141 heteroduplex.

The study examining the thermal stability of PNA-miR141/miR141 heteroduplexes provided clearer results (see Figure S7).

Specifically, *T*_m_ = 75.3 °C was measured for the PNA-miR141/miR141 heteroduplex in PBS without Mg^2+^. When 0.3 mM MgCl_2_ was added to the PBS buffer, there was a slight increase in the thermal stability, resulting in a *T*_m_ of 79.3 °C. However, further increases in MgCl_2_ concentrations (30 and 100 mM) did not lead to an additional rise in the *T*_m_ values. The 4 °C increase in *T*_m_ observed with a Mg^2+^ concentration of 0.3 mM or higher does not adequately account for the significant enhancement of the measured SPRI signal. Furthermore, the independence of *T*_m_ from Mg^2+^ concentration within the 0.3 to 100 mM concentration range further indicates that the observed effect of Mg^2+^ on surface-confined PNA/miRNA hybridization does not occur in similar hybridizations in solution.

## 4 Conclusions

Despite the widespread use of PNAs as capture probes for targeting nucleic acids, there is still a limited understanding of the interactions between surface-bound PNA and miRNA targets. Additionally, the impact of reaction conditions, such as electrolyte concentrations, on the efficiency of miRNA target capture is not fully understood. For this reason, we investigated two different PNA–miRNA interactions using the targets miR125 and miR141 by employing SPRI.

Our SPRI experiments revealed that the hybridization of miRNA with complementary surface-immobilized PNA probes is significantly enhanced when Mg^2+^ is added to the PBS buffer at concentrations ranging from 30 to 100 mM. Further analysis of the kinetics of the association and dissociation reactions showed that 30 and 100 mM concentrations of Mg^2+^ facilitate the association between the immobilized PNA probe and the complementary miRNA target. This effect likely arises from the effective screening of the negatively charged backbones of the miRNA molecules as they approach the surface. Additionally, these Mg^2+^ concentrations stabilize the heteroduplexes formed on the surface, significantly reducing the dissociation rate. Higher Mg^2+^ concentrations (300 mM) hindered the surface-confined hybridization.

The experimental observations and considerations outlined above offer insights into the factors that contribute to the differing binding strengths observed in the interaction between PNA and miRNA. These insights are particularly relevant for miRNA biosensing, as they can directly affect critical aspects of biosensors such as sensitivity and measurement reliability.

Changes in miRNA expression levels, whether upregulated or downregulated, can provide critical information about numerous diseases including cancer. As a result, targeting miRNAs is becoming increasingly important in both therapeutic and diagnostic applications. Enhancements in the performance of SPRI sensors for miRNA detection, when coupled with PNA probes and optimized experimental conditions, have the potential to overcome common challenges associated with analyzing very small amounts of target material in biofluids. This improvement could dramatically enhance the reaction efficiency in miRNA detection, leading to significant applications in both research and clinical settings.

## Supplementary Material



## Data Availability

All data needed to evaluate the conclusions in the paper are present in the paper and/or the Supporting Information.

## Notes

SPR imaging measurement details and data; melting analysis; PNA characterization; sequences used in this study; and parameters obtained after hybridization of miR125 with immobilized PNA-miR125 using PBS buffer and various ion concentrations (PDF)
