# Supplementary material for: Peptide Nucleic Acid Probes for MicroRNA Detection: Mg2+ Ion Effect, Surface Hybridization, and Surface Plasmon Resonance Biosensing
Source: ACS Meas Sci Au. 2025 Oct 21;5(6):868–78. doi: 10.1021/acsmeasuresciau.5c00100 (PMC12715733; doi:10.1021/acsmeasuresciau.5c00100)
Supplement: Supplementary file 1 [file tg5c00100_si_001.pdf]

Supporting Information

Peptide Nucleic Acid Probes for MicroRNA Detection: Mg^{2+} ions Effect, Surface Hybridization, and Surface Plasmon Resonance Biosensing

Vanessa Jungbluth[1†], Roberta D'Agata[1,2†], Noemi Bellassai[1,2], Stefano Volpi[3], Roberto Corradini[2,3], Giuseppe Spoto[1,2*]

[1] Department of Chemical Sciences, University of Catania, Viale Andrea Doria 6, 95122, Catania, Italy.
[2] INBB, Istituto Nazionale di Biostrutture e Biosistemi, Via dei Carpegna 19, 00165 Roma, Italy.
[3] Department of Chemistry, Life Sciences and Environmental Sustainability, University of Parma, Parco Area Delle Scienze, 17/A, 43124, Parma, Italy.

*Corresponding author: email: giuseppe.spoto@unict.it

[†]Contributed equally to this work

SPR imaging measurements....................2
Melting analysis....................7
PNAs characterization....................10
Table S1....................11
Table S2....................12
Table S3....................13

SPR imaging measurements

All SPRI experiments were carried out using an SPR imager apparatus (GWC Technologies, USA). SPR images were analyzed using V++ software. The obtained SPRI data provided data as pixel intensity units (0-255 scale), which was converted into a percentage of reflectivity (%R) or in Δ%R in the case of difference images, by using the formula:

$$\%R = 100\,x\,\frac{0.85Ip}{Is}$$

Where I_p and I_s refer to the reflected light intensity detected using p- and s-polarized light, respectively. Experiments were carried out by sequentially acquiring 15 frames averaged SPR images with a 5 s time delay between them. Kinetic data were obtained by plotting the difference in percent reflectivity (Δ%R) from selected regions of interest (ROIs) of SPR images as a function of time. The selected ROIs were chosen to include all the SPR chip areas involved in the surface interaction experiment. All the SPRI experiments were carried out at room temperature. A microfluidic device with six parallel microchannels (200 µm depth, 1.4 cm length, 400 µm width) and circular reservoirs (diameter = 400 µm) at the ends of each channel was used for the study to allow independent control of interactions occurring on six different regions of the SPRI gold chip surface. The device was fabricated in poly(dimethylsiloxane) (PDMS) polymer using the replica molding technique. PEEK tubes (UpChurch Scientific) were inserted in the circular reservoirs to connect the PDMS microfluidic cell to an Ismatec IPC (Ismatec SA, Switzerland) peristaltic pump. The microfluidic device was assembled by fixing the PDMS mold on the SPRI gold chip surface. A refractive index matching liquid was used to obtain the optical contact between the gold chip and the prism. A strict cleaning procedure of the fluidic system was adopted to minimize contaminations and memory effects. The fluidic system was washed with ultraclean water (37 °C, for 2 h) after each experiment. Every 3 weeks the system was cleaned as follow: 0.5% sodium dodecyl sulfate (SDS) (10 min), 6 M urea (10 min), 1% acetic acid (10 min), 0.2 M $NaHCO_3$ (10 min), ultraclean water 37 °C (30 min) and PBS buffer. Gold chips (Xantex, Germany) were functionalized with DTSP before the immobilization of PNA probes. We obtained DTSP functionalization as follows: a bare gold chip was treated with UV/ozone for 5 min, then washed with ethanol for 10 min and dried under a nitrogen stream. The cleaned chip was immersed for 48 h in DTSP solution (4 mM in DMSO) under constant and gentle agitation.

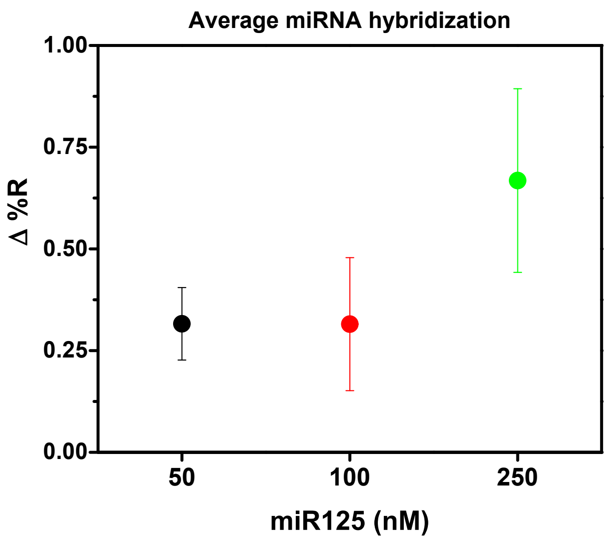

Figure S1: Average Δ%R for hybridization of miR125 (50, 100 and 250 nM; in black, red, and green, respectively) to a PNA-miR125 immobilized on the sensor surface. Error bar represents the standard deviation (n>3).

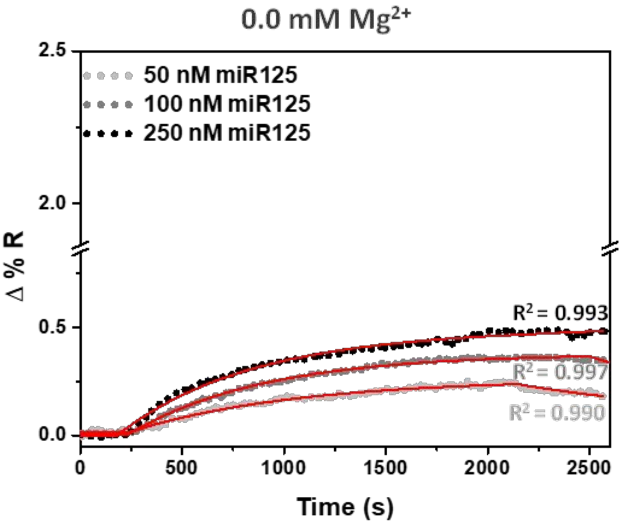

Figure S2. Representative SPRI hybridization curves between PNA-miR125 and miR125 at different concentrations of 50 nM (light grey dots), 100 nM (dark grey dots), and 250 nM (black dots) in PBS buffer without Mg^{2+}. Fitting curves (red lines) were obtained by Anabel software to estimate rate constants. The coefficient of determination, R^2, calculated for each fitting curve at different miRNA concentrations, was reported.

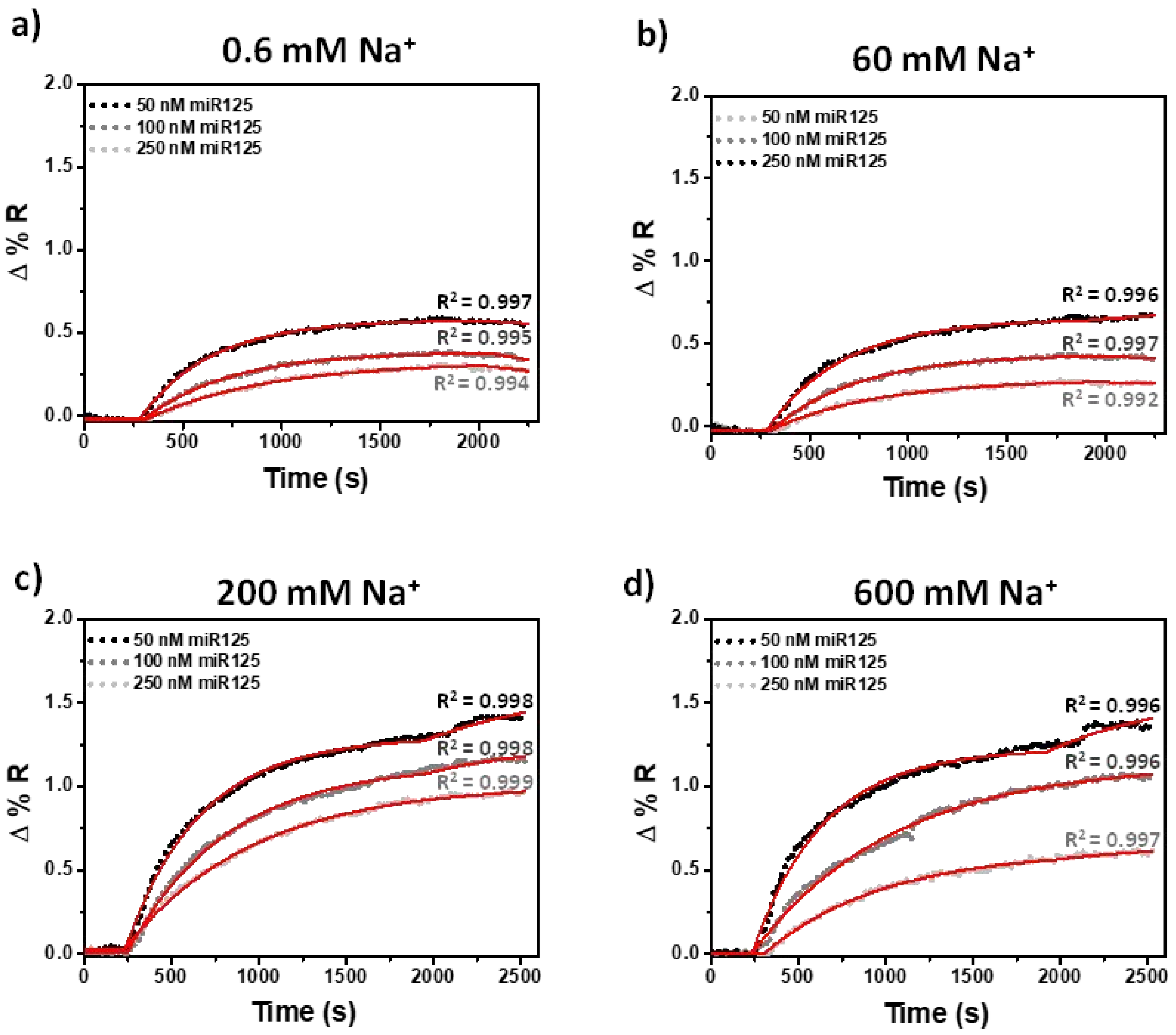

Figure S3: Representative SPRI hybridization curves between PNA-miR125 and miR125 at different concentrations of 50 nM (light grey dots), 100 nM (dark grey dots), and 250 nM (black dots) in PBS buffer with 0.3 mM (a), 60 mM (b), 200 mM (c), and 600 mM (d) of Na^+. The red lines are the global nonlinear least squares fit obtained by Anabel software to estimate the affinity constants with different Na^+ concentration (Table S2). The coefficient of determination, R^2, was calculated for each fitting curve at different miRNA concentrations.

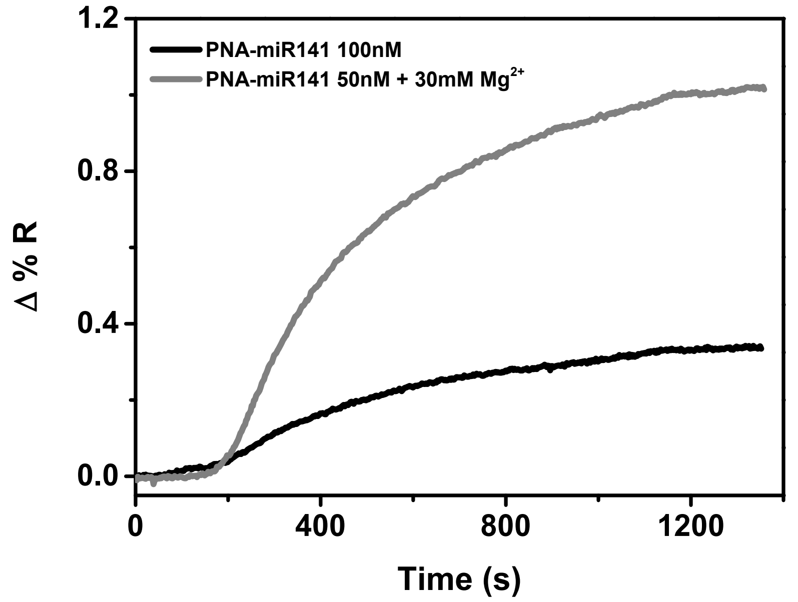

Figure S4: Δ%R for the SPRI hybridization reaction between PNA-miR141 immobilized onto the gold surface and miR141 (50 nM in PBS added with 30 mM $MgCl_2$, grey line) and miR141(100 nM in PBS and no $MgCl_2$, black line).

Figure S4 illustrates the SPRI responses observed when a 100 nM miR141 solution in PBS (black line) interacts with a surface-immobilized PNA-miR141 probe. This is compared to a 50 nM miR141 solution in PBS that also includes 30 mM $MgCl_2$ (grey line). The SPRI signal detected after the adsorption of the 50 nM miR141in the presence of Mg^{2+} was nearly three times greater than the signal detected with the higher concentrated 100 nM miR141 solution without Mg^{2+}.

Melting analysis

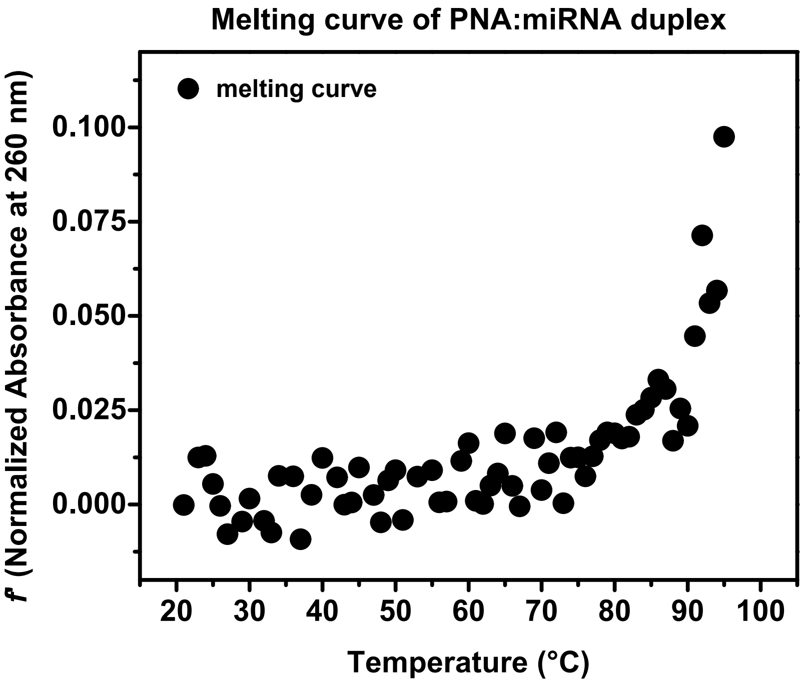

Figure S5: Normalized melting curve of the PNA-miR125 heteroduplex represented as normalized absorbance versus temperature (λ =260 nm). Attempts to determine the melting temperature (Tm) of the PNA-miR125:miR125 heteroduplex by monitoring UV absorbance at 260 nm as a function of temperature were unsuccessful due to its exceptionally high stability. In fact, although a sigmoidal trend was observed at the upper end of the explored temperature range, the inflection point of the melting curve lays beyond 95°C. Similar results were obtained using circular dichroism (CD) spectroscopy (data not shown). These findings are consistent with a calculated T_m of 83.4 °C for a homologous PNA–DNA heteroduplex (Table S1), due to the higher stability of the corresponding PNA–RNA duplex.

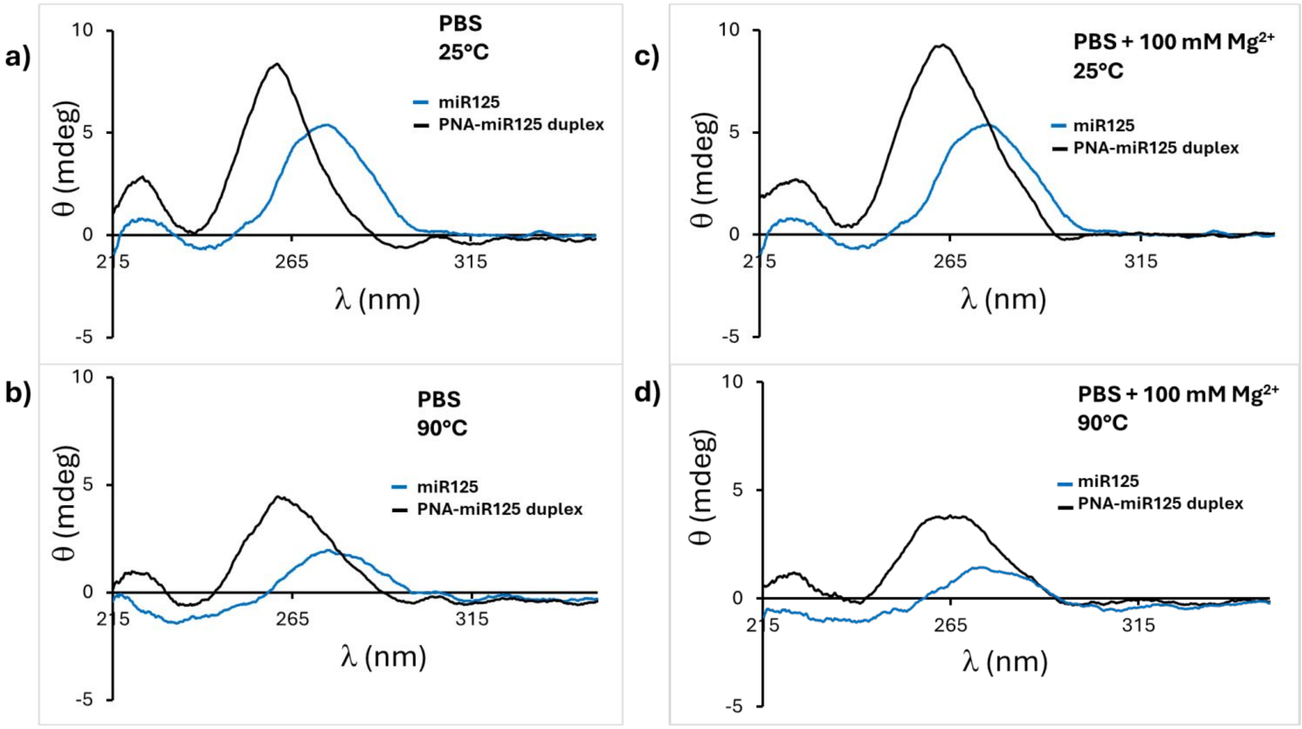

Figure S6. CD spectra of miR-125 (blue lines) and PNA-miR125:miR-125 heteroduplex (black lines) recorded at **a)** 25°C in PBS K812 buffer, **b)** 25°C in PBS K812 buffer + 100 mM $MgCl_2$, **c)** 90°C in PBS K812 buffer, **d)** 90°C in PBS K812 buffer + 100 mM $MgCl_2$. The strands concentration was 5 μM for the free RNA and 4.8 μM for the PNA:RNA duplex. Cell length was 0.2 cm.

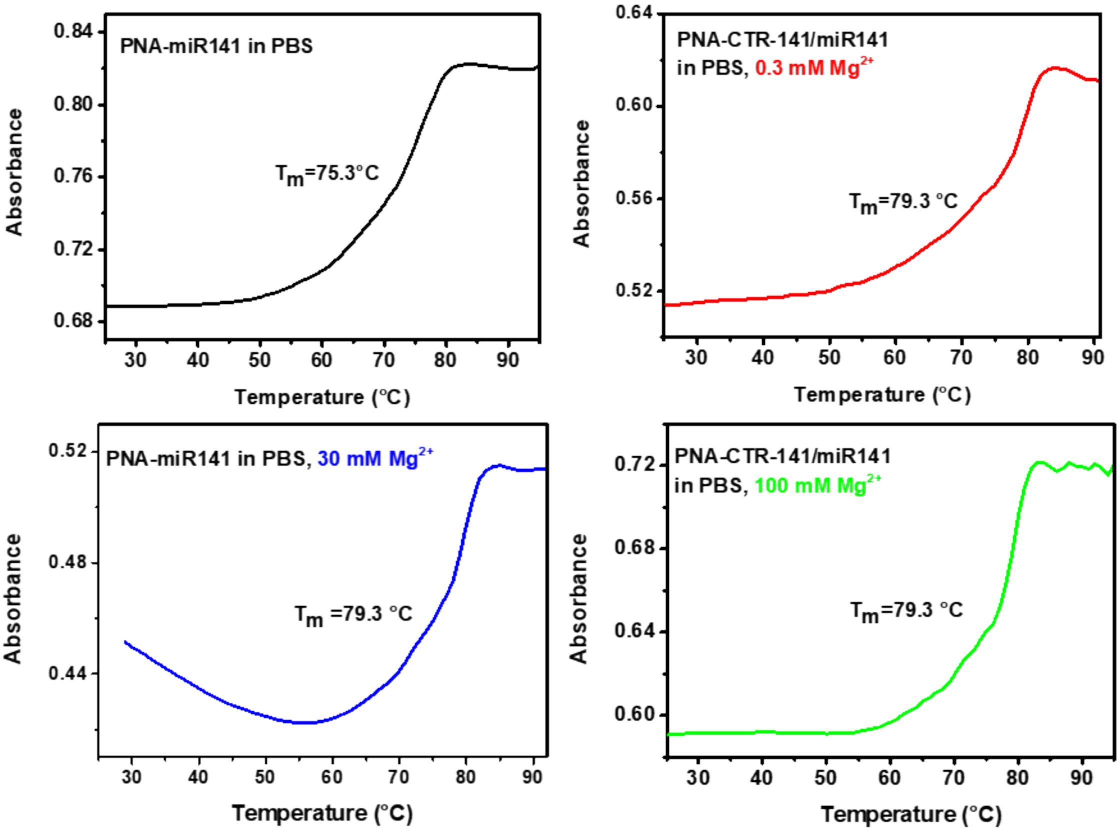

Figure S7: Melting temperature curves for PNA-miR141 and miR141 (2uM) in PBS (black curve), in PBS, 0.3mM Mg^{2+} (red curve), in PBS 30mM Mg^{2+} (blue curve), in PBS 100 mM Mg^{2+} (green curve).

PNAs characterization

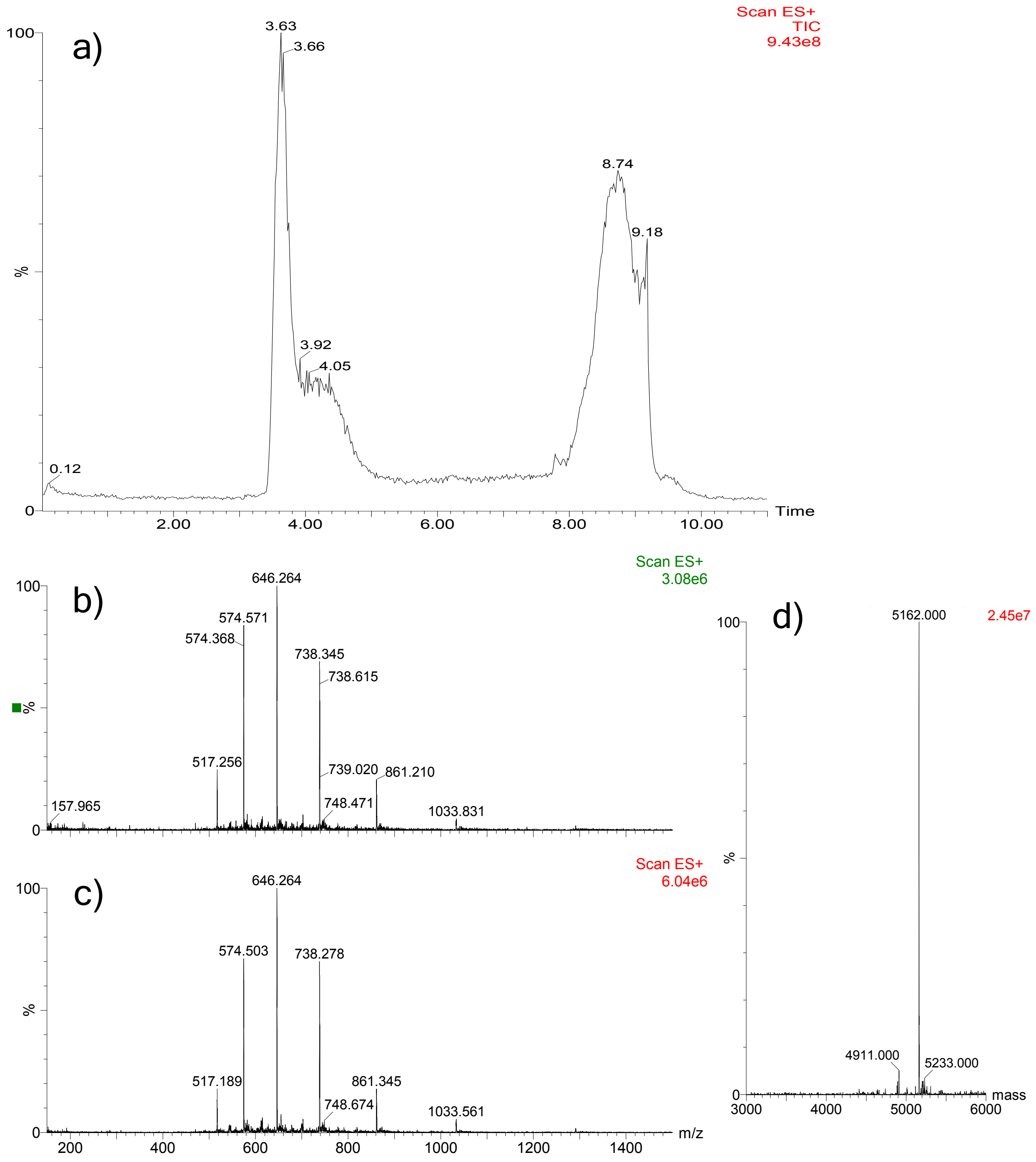

Fig. S8: UPLC-MS (ESI-MS, positive mode, water) chromatogram of pure PNA-miR141 (top) and ESI-MS spectrum for the peak at b) 4.02 min and c) 3.63 min with the corresponding mathematical deconvolution of the multicharged signals (d). Both peaks have the same MS spectrum and were therefore attributed to differently folded forms of the product, corresponding to the following data:

Calculated MW for H-O-O-CCATCTTTACCAGACAGT-Gly-NH_2: 5162.09 [M]; m/z found (calcd) 1033.6 (1033.4) $[M+5H]^{5+}$, 861.3 (861.3) $[M+6H]^{6+}$, 738.3 (738.4) $[M+7H]^{7+}$, 646,3 (646.2) $[M+8H]^{8+}$, 574.5 (574.6) $[M+9H]^{9+}$, 517.2 (517.2) $[M+10H]^{10+}$.

Table S1: Sequences used in this study and corresponding calculated melting temperatures. Underlined letters identify miRNA sequences complementary to the corresponding PNA probe.

Acronym	Sequence*	Calculated Tm (°C)
**PNA-miR125**	Ac-AAG TTA GGG TCT CAG GGA-Lys(AEEA)$_2$-NH$_2$	83.4 **
**PNA-miR141**	H-(AEEA)$_2$-CCA TCT TTA CCA GAC AGT-Gly-NH$_2$	73.1**
**miR125**	UCC CUG AGA CCC UAA CUU GUG A	65.4 #
**miR141**	UAA CAC UGU CUG GUA AAG AUGG	61.6 #

*PNAs included two (2-(2-aminoethoxy)ethoxy acetyl)) (AEEA) spacers at C-term or N-term. **PNA/DNA melting temperatures calculated at 4 µM strand concentration using the on-line tools PNA-BIO (http://pnabio.com/support/PNA_Tool.htm); #DNA/DNA melting temperatures for the PNA targeted sequence calculated at 4 µM strand concentration and 0.1M salt concentration by Multiple Primer Analyzer Tool from ThermoFisher (https://www.thermofisher.com/it/en/home/brands/thermo-scientific/molecular-biology/molecular-biology-learning-center/molecular-biology-resource-library/thermo-scientific-web-tools/multiple-primer-analyzer.html), respectively.

Table S2 : Parameter obtained after the hybridization of miR125 to PNA-miR125 immobilized to the sensor surface using PBS hybridization buffer without and with 0,3, 30, 100, 300 mM Mg^{2+} added. For the thickness and surface coverage, we used a density of 1.89 g cm^{-3} and a refractive index of 1.48 for RNA sequences.[1,2]

[miR125] (nM)	[$MgCl_2$] (mM)	Reflectivity change (Δ%R±SD)	Average thickness of the surface layer d (nm)	Surface coverage (θ) (molecules cm^{-2}) × 10^{12}	Hybridization efficiency HE (%)	K_A* (M^{-1})
50	0	0.32 ± 0.13	0.05	0.91	16	3.18 ±1.46x10^6
100		0.31 ± 0.16	0.05	0.88	16	
250		0.67 ± 0.23	0.11	1.90	34	
50	0.3	0.29 ± 0.16	0.05	0.82	14	4.67±1.53x10^6
100		0.42 ± 0.15	0.07	1.22	22	
250		0.57 ± 0.21	0.10	1.66	30	
50	30	1.10±0.14	0.19	3.11	56	1.93 ± 0.25x10^7
100		1.19±0.19	0.20	3.37	60	
250		1.55±0.19	0.26	4.39	78	
50	100	1.09±0.40	0.19	3.09	55	3.4±0.08x10^7
100		1.40±0.32	0.24	3.96	71	
250		1.95±0.25	0.33	5.52	98	
50	300	0.86±0.15	0.15	2.43	43	2.04±0.63x10^6
100		1.07±0.07	0.18	3.03	54	
250		1.01±0.12	0.17	2.86	51	

*K_A calculated considering the k_{on} and k_{off} values calculated from SPRI measurements reported in Figure 3a.

Table S3: Parameters obtained for the hybridization of miR125 to PNA-miR125 immobilized to the sensor surface using PBS added with various Na^+ concentrations (0.6, 60, 200, 600 mM).

[miR125] (nM)	[NaCl] (mM)	Reflectivity change (Δ%R±SD)	Average thickness of the surface layer d (nm)	Surface coverage (θ) (molecules cm^{-2}) × 10^{12}	Hybridization efficiency HE (%)	K_A* (M^{-1})
50	0.6	0.27	0.04	0.62	11	3.93±2.06 x 10^6
100		0.33	0.05	0.73	13	
250		0.55	0.09	1.53	27	
50	60	0.26	0.15	2.53	45	5.8±2.66 x 10^6
100		0.41	0.18	2.99	53	
250		0.67	0.10	1.70	30	
50	200	0.98	0.15	2.53	45	4.96±3.72 x 10^6
100		1.16	0.18	2.99	53	
250		1.42	0.24	3.94	70	
50	600	0.62	0.11	1.85	33	3.51±0.56 x 10^6
100		1.08	0.17	2.83	51	
250		1.38	0.22	3.58	64	

* K_A calculated considering k_{on} and k_{off} values calculated from SPRI measurements reported in Figure 5.

---

[1] C MT Spahn, P A Penczek, A Leith, J Frank, A method for differentiating proteins from nucleic acids in intermediate-resolution density maps: cryo-electron microscopy defines the quaternary structure of the Escherichia coli 70S ribosome, Structure 8, 2000, 937-948.

[2] S Venkatasubbarao, N Beaudry, Y Zhao, R Chipman, Evanescent-imaging-ellipsometry-based microarray reader, Journal of Biomedical Optics 11, 2006, 014028.
